# Temperature-Independent Fiber Inclinometer Based on Orthogonally Polarized Modes Coupling Using a Polarization-Maintaining Fiber Bragg Grating

**DOI:** 10.3390/s141120930

**Published:** 2014-11-05

**Authors:** Dan Su, Xueguang Qiao, Hangzhou Yang, Qiangzhou Rong, Zhengyuan Bai, Yupeng Wang, Zhongyao Feng

**Affiliations:** 1 School of Physics, Northwest University, Xi'an 710069, China; E-Mails: xgqiao@nwu.edu.cn (X.Q.); yanghz@nwu.edu.cn (H.Y.); qzrong2010@gmail.com (Q.R.); ypgood@163.com (Y.W.); rqznwu@gmail.com (Z.F.); 2 Key Laboratory of Material for High Power Lasers, Shanghai Institute of Optics and Fine Mechanics, Chinese Academy of Sciences, Shanghai 201800, China; E-Mail: zybai@siom.ac.cn

**Keywords:** fiber optics sensor, polarization-maintaining fiber bragg grating, bending

## Abstract

A reflection fiber inclinometer is proposed and experimentally demonstrated based on two linearly polarized (LP) modes coupling. The configuration consists of a section of polarization-maintaining fiber (PMF) containing a fiber Bragg grating (FBG) splicing with single mode fiber (SMF). Bending the PMF in the upstream of FBG can induce an additional birefringence of PMF, which results in the intensity changes of two LP modes owing to orthogonal polarization coupling. The experimental results represent that the device shows different bending responses at the angle range from 0° to 40°and from 64° to 88°, respectively. Moreover, the temperature change just shifts the wavelengths of LP modes reflected and does not influence their intensities, which effectively avoid the temperature cross-sensitivity and make it a good candidate for measuring inclinometer and temperature simultaneously.

## 1. Introduction

Fiber Bragg Gratings (FBGs) inscribed in single mode fiber (SMF) have been widely used in communications and sensing fields due to their advantages of small size, light weight, and narrow filter bandwidth. Diverse FBG-based sensors have been proposed for measuring temperature [1], strain [2] and vibration [3] by monitoring the wavelength shifting or bandwidth broadening of reflection spectrum. Besides, FBGs inscribed in diverse polarization-maintaining fiber (PMFs) outperform traditional FBGs in responding asymmetrical perturbation within fiber due to the intrinsic property of PMF. PMFs, such as Panda-type PMF, bow-tie type PMF and elliptical core PMF as the generations of optic fibers present the intrinsic high-birefringence due to their circular-asymmetric cross-section structure. Their polarimetric property which is sensitive to anisotropic disturbance inside PMF has been employed as interferometer [4], and to equip the fiber-optic gyroscopes [5], and lasers [6].These devices are normally based on the interference between orthogonal modes. FBG written in the PMF (PM-FBG) reflects two orthogonal modes along the two principal axes (the fast- and slow-axis of PMF) with respect to the different wavelengths. The perturbation implemented in PM-FBG, such as shear strain [7], torsion [8], and stress [9] can provide an additional birefringence in the PMF, which breaks the original polarization coupling and builds a new E-field distribution along the two principal axes. However, the accompanied chirp of grating and temperature cross-sensitivity will disturb the signal measurement.

Bending is an important physical parameter due to its multi-area applications including security monitoring and structure engineering. Fiber-optic inclinometers for measuring bending angle have been developed in various schemes, such as in-line Mach-Zehnder interferometer by concatenating two low-loss fused tapers [10], polarization-maintaining crystal fiber (PM-PCF)-based inclinometer [11], which is a wavelength-referenced and has the unwanted temperature cross-sensitivity, and FBG combined with an etched SMF [12]. Although these smart devices present sensitive response to the bending angle, they suffer from some downsides of temperature cross-sensitivity and weakened mechanical structure (due to etching).

In this paper, we propose a reflection-type fiber inclinometer with a PM-FBG spliced with a lead-in fiber. The coupling between LP_01_(*x*) and LP_01_(*y*) modes of PMF and bending-induced loss are employed to demonstrate the bending angle at a large range from 0° to 90°. At the measured angle range from 0° to 40°, the device presents high orientation-dependence because of the LP coupling. In the sensing mechanism, the E-field changes of the orthogonal LP modes are demonstrated, which can avoid the temperature cross-sensitivity.

## 2. Schematic Diagram and Principle of the Sensor

**Figure 1.**
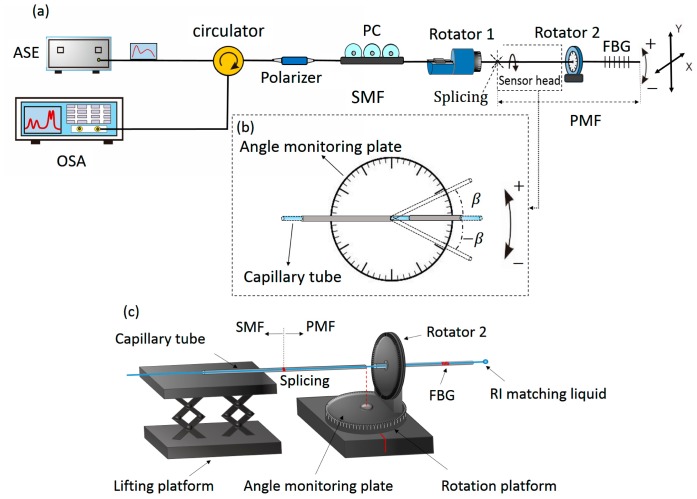
(**a**) Experimental system for bending sensing; (**b**) schematic diagram of the bending angle; (**c**) simulated setup of the inclinometer function.

Figure 1 shows the schematic diagram of the bending sensing system. The light is emitted from an amplified spontaneous emission (ASE, LIGHTCOMM, intensity errors are ≤±0.005 dB which can be compensated using a splitter and connecting the optical source directly to a photodiode as [13]) and propagates through a polarizer to obtain a highly linear polarized light. A commercial polarization controller (PC) following with the polarizer is used to adjust the polarization state of the light. The polarized light is launched into the sensing fiber of PMF and then reflected by FBG downstream. An optical spectrum analyzer (OSA) with a resolution ratio of 0.01 dB is employed to demonstrate the spectra variation, as shown in the Figure 1a. In order to eliminate the unwanted background noise caused by the fiber tip reflection, the end face of the PM-FBG downstream is coated with the refractive index (RI) matching liquid. The sensing device consists of a 14 cm-long bow-tie type PMF (containing a 12 mm-long FBG) which is spliced with a lead-in standard SMF by using a commercial fusion splicer (Fujikura FSM-60S) with the standard arc discharge condition.

When the high linearly polarized light is launched into the PM-FBG, two polarized modes of LP_01_(*x*) and LP_01_(*y*) are generated and transmit along the slow- and fast-axis of PMF, and then reflected by FBG which is used as a polarization-sensitive reflector. The intrinsic birefringence of PMF is:

(1) $$B=\left|\beta_{\text{x}}-\beta_{\text{y}}\right|=\frac{2\pi }{\lambda }\left(n_{eff}^{x}-n_{eff}^{y}\right)$$

where the two LP modes present different propagation constants of β_x_ and β_y_, the *nxeff* and *nyeff* are the effective refractive indices of the slow- and fast-axis of PMF. The E-field intensities of two LP modes of PMF can be written as

(2) $$E_{x}=A_{0}\cos\theta \sin\left(\omega t-\beta_{x}z\right)$$

(3) $$E_{y}=A_{0}\sin\theta \cos\left(\omega t-\beta_{y}z\right)$$

where the *A*_0_ is the original amplitude of the E-filed, and θ is the angular orientation of the linearly polarized incident light. When the small bending is applied on the short section of PMF, the anisotropic strain deforms the geometrical cross-section of PMF, which changes the refractive index of the fused silica due to the photoelastic effect. The PMF at the inner side of the bending axis are compressed and that at the outside is stretched, and thus compels the refractive index of fused silica to decrease at one side and increase at the opposite side. As a result, the bending induces an additional linear birefringence which will overlap the intrinsic birefringence of PMF. Consequently, the bending angle changes the orthogonal polarized modes coupling, resulting in the redistribution of the intensities of the resonant modes. At the bending *x*-*y* plane (*i.e.*, the stress of *z* axis is 0), the bending-induced birefringence change between polarized-modes can be expressed in the form:

(4) $$\delta B=0.25kn^{3}\left(P_{11}-P_{12}\right)\left(1-\mu \right){\left(R_{0}\cos\varphi \right)}^{-2}r^{2}$$

(5) $$B\left(\beta \right)=B_{0}+\delta B$$

where *k* = 2π/λ, *n* is the refractive index of the fiber core, *P*_11_ = 0.12 and *P*_12_ = 0.27 are the strain-optical coefficient, μ = 0.17 is the Poisson's ratio, R_0_ represents the original bending, φ is the bending angle, and *r* is the radius of the PMF. According to the Equation (4), the adding birefringence of PMF significantly depends on the φ. In addition, with the increasing of the bending angle, the bending-induced loss will play a main role, and the intensities of two LP modes decrease simultaneously. The curvature loss of PMF can be written as [14]:

(6) $$2\alpha =\frac{\sqrt{\pi }K^{2}\exp\left[-\frac{2\gamma^{3}}{3\beta^{2}}R\right]}{2e_{V}\gamma^{3/2}V^{2}\sqrt{R}K_{1}^{2}\left(\gamma a\right)}$$

where *a* is the radius of the fiber core, and the parameters *K*, γ, *V* are defined as:

$$\begin{array}{ll} K & ={\left(n_{1}^{2}k^{2}-\beta_{0}^{2}\right)}^{1/2}\\ \gamma & ={\left(\beta_{0}^{2}-n_{1}^{2}k^{2}\right)}^{1/2}\\ V^{2} & =a^{2}\left(K^{2}+\gamma^{2}\right) \end{array}$$

where β_0_ is the unperturbed propagation constant of fundamental mode of the straight fiber. The transmission loss of PMF linearly functions as the fiber bending at the range of 13.5 to 24 mm [15].

## 3. Experiment and Discussion

**Figure 2.**
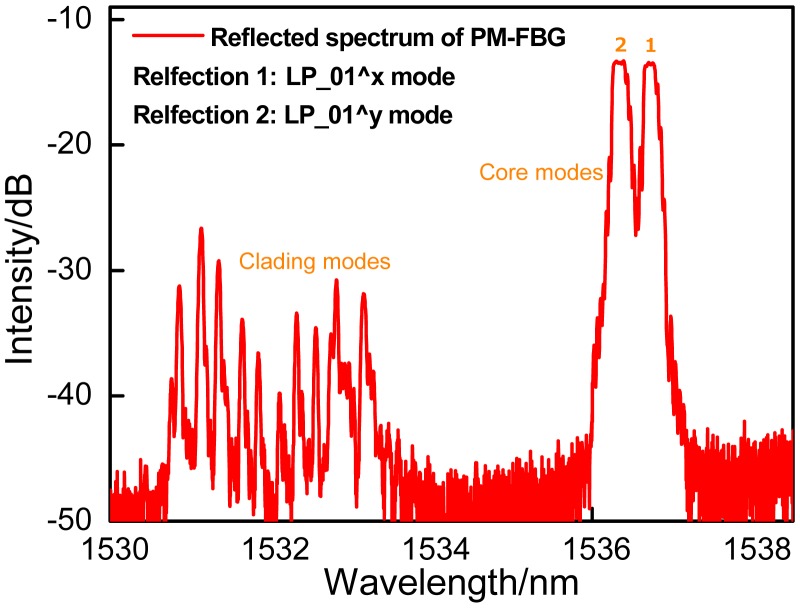
Experimentally measured reflection spectrum of sensing PM-FBG.

The schematic diagram of the bending sensing system has been shown in the Figure 1a. Figure 1b,c is the schematic diagrams of the inclinometer function. The SMF upstream is fixed onto the rotator 1, and the PMF is glued onto the rotator 2. The rotator 2 is attached onto another rotation platform functioning to bend the PMF. Two sections of capillary tubes are employed to coat the fiber to keep the both sides of fiber straight, while just a few centimeters of the PMF at the middle of tubes is utilized to respond to the bending. The bending section of PMF must be set above the center of the rotation platform. When the platform is rotated, the rotator 2 makes the bare PMF bend. Besides, as shown in the Figure 1b, the rotation platform is also utilized as an angle monitoring plate to calibrate the bending angle. In our prior work, polarization-maintaining photonics crystal fiber (PM-PCF) with 1 cm, 1.5 cm and 2 cm-long bending sections have been used to measure the bending [11],of which the longer bending section has a decreased bending sensitivity, since it weakens the phase difference accumulation and bending loss at the same bending angle, compared with the shorter one. In the experiment, 1 cm-long PMF is employed as a case, which can ensure the device to present a good sensitivity. The reflection spectrum of the device is shown in the Figure 2. It can be seen that the usual pair of resonances corresponding to the two orthogonal polarized states of the core mode of PMF and a group of high-order polarized cladding modes are obtained. Although the high-order modes are more sensitive to the fiber bending than the core mode, their low intensities significantly limit the bending measurement range. The initial E-field intensities of LP_01_(*x*) and LP_01_(*y*) modes are equal when the LP light is launched into the PMF at 45° with respect to each birefringence axis of PMF by regulating the polarization controller.

**Figure 3.**
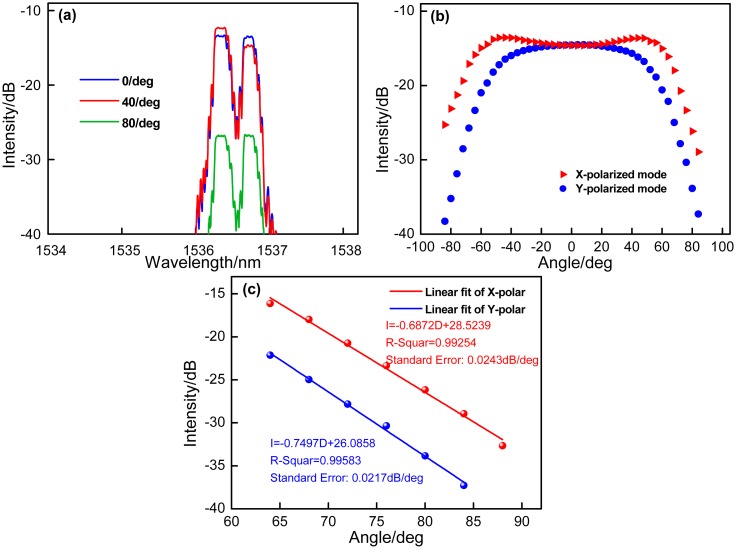
(**a**) Change of coupling intensity the LP01(*x*) and LP01(*y*) modes with the different bending angle; (**b**) intensities of *x*- and *y*-polarized modes act as the function of the bending angle; (**c**) linear fit of intensity of the *x*- and *y*-polarized mode changes with the bending angle at the range from 64° to 88°.

**Figure 4.**
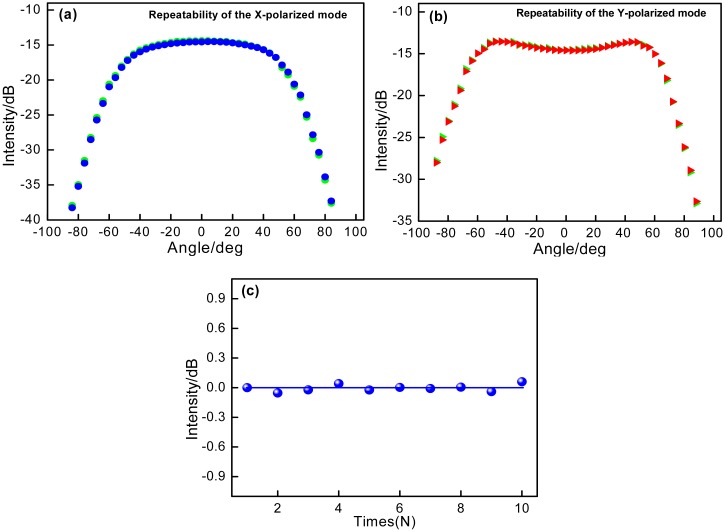
Repeatedly testing of the intensity responses to angles of (**a**) *x*-polarized mode; (**b**) *y*-polarized mode; and (**c**) quantitative repeatability statement at the same bending angle.

When the bare 1cm-long PMF is bended, the coupling of the LP01(*x*) and LP01(*y*) modes occurs, as the blue and red lines shown in Figure 3a. It is seen that increasing the bending angle results in the intensities of the two reflected resonant wavelengths trading off and taking turns initially. When the PMF is bended to a large angle, the intensities of the two resonant wavelengths decrease simultaneously, as the green line shown in Figure 3a, which is attributed to that the bending-induced loss mainly determines the intensities of the resonant modes at the large bending angle of PMF. Figure 3b demonstrates the intensities of *x*- and *y*-polarized modes changes with the bending angle at the range from 0° to +88°/−88° with a step of 4°. At the initial bending angle ranging from 0° to 40°, the coupling between *x*-and *y*-polarized core modes plays a key role, and thus the intensities of two LP modes present opposite change. With sequentially increasing the bending angle from 40° to 88°, the bending-induced loss of PMF will mainly determine the intensities of two LP modes. At the angle range from 64° to 88°, the bending-induced loss will linearly decrease the intensities of both LP modes, and two sensitivity values of −0.6872 dB/deg and −0.7497 dB/deg with the linear fitting coefficients of 0.99254 and 0.99583 are obtained respectively, as shown in Figure 3c, and the minimum detectable angle variation is 0.014°. Moreover, the standard errors are analyzed of 0.0243 dB/deg and 0.0217 dB/deg. In addition, to confirm the sensing repeatability of the proposed device, the *x*- and *y*-polarized modes' responses to bending angles are implemented repeatedly at the certain polarization-orientation controlled by two rotators. As shown in Figure 4, two LP modes present good repeatability. In Figure 4c, when bending the sensor head to the same angle over 10 times, the results show a high repeatability, and the intensities is just presented as the slight fluctuations about 0.026 dB.

**Figure 5.**
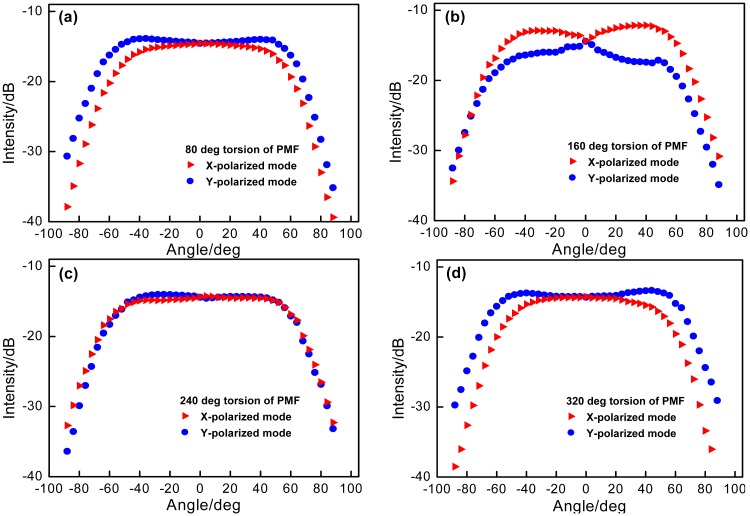
Device's responses to bending angle at the different rotation orientations of PMF, (**a**) 80°; (**b**) 160°; (**c**) 240° and (**d**) 320°.

Due to the important character of the E-field vector [16,17], at the measured angle ranging from 0° to 40°, the orientation-dependence of the device has also been tested. The rotators 1 and 2 are employed to rotate the PMF and rotated simultaneously to the same angle of 80°, 160°, 240° and 320° (the rotation orientation of the PMF in the initial experiment is considered as 0°) for avoiding the PMF twist. Figure 5a–d demonstrates the corresponding bending angle responses of the device at different rotation orientations. The device presents distinctly different sensitivities to the bending angles, especially at the range from 0° to +/−40° as a “polarization-sensitive region”. Among them, the larger sensitivity is as the result of that the bending axis is parallel to the polarization axis, and the smaller one is due to that each polarization is independent of the bending occurring in the orthogonal direction. Generally, at each rotation orientation of PMF, the bending-induced loss response sensitivities of the device should be the same. However, the influence of the intrinsic and additional birefringence changes will overlap that of the bending loss, which may disturb the bending-induced loss response sensitivities at different bending operation orientations.

**Figure 6.**
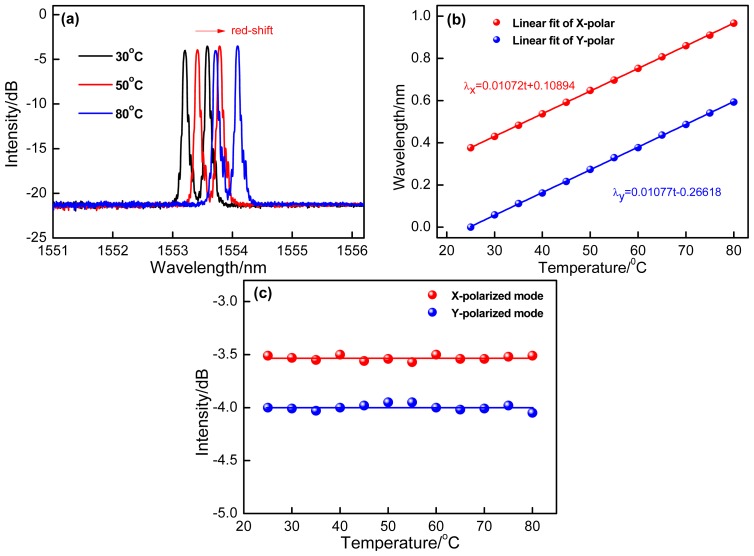
(**a**) Experimentally measured reflection spectra of the *x*- and *y*-polarized modes which shows red-shift over the heating process; (**b**) The reflection wavelengths versus temperature; (**c**) Their corresponding intensities fluctuations.

The temperature response of the proposed sensor has also been characterized. As shown in the Figure 6a, over the heating process at the range from 25 °C to 80 °C, the reflection spectra typically shows red-shift. In Figure 6b, we plot the reflection wavelengths function as the increasing temperature with extremely linear sensitivities of 10.72 pm/°C and 10.77 pm/°C with respect to two polarized modes. At the same time, the intensities of two polarized modes versus temperature are shown in Figure 6c. It is seen that their intensities just present the slight fluctuations (±0.04 dB to *x*-polarization and ±0.05 dB to *y*-polarization). Compared with the bending sensitivities of two polarized modes, the intensities fluctuations resulting from temperature are so small and can be ignored, so the bending measurement is independent of temperature.

## 4. Conclusions

A reflection-type fiber inclinometer with a PM-FBG as the polarimetric mirror is experimentally demonstrated to measure the bending angles. A polarizer and a polarization controller are used to controller the prior polarization of light launching into the PM-FBG. A short section of upstream PMF close to the FBG is as bending region to respond the angles changes ranging from 0° to 88°. Moreover, the reflected intensities of sensor response are immune to temperature fluctuations of several tens of degrees Celsius. Its large-angle sensing range and the high bending sensitivity will make this device a candidate for measuring inclinometer and temperature simultaneously.
